# From Lifespan to Healthspan: Integrating Nutrition and Physical Activity in Healthy Ageing

**DOI:** 10.3390/nu18030528

**Published:** 2026-02-05

**Authors:** Bartłomiej K. Sołtysik

**Affiliations:** Department of Geriatrics and Internal Medicine, Healthy Ageing Research Centre (HARC), Medical University of Lodz, Pomorska Street 251, 92-213 Lodz, Poland; bartlomiej.soltysik@umed.lodz.pl

Population ageing is no longer a future scenario but a present reality, reshaping not only the epidemiology of chronic disease but also the fundamental goals of medicine and public health [1]. As life expectancy continues to increase, the central challenge is no longer how to live longer, but how to live longer while preserving physical function, independence, and quality of life [2]. In this context, lifestyle-related factors, particularly diet and physical activity, stand out as among the most accessible and most powerful determinants [3]. Yet, despite decades of accumulating evidence, the burden attributable to modifiable lifestyle risks remains strikingly high, with dietary factors still ranking among the leading contributors to global morbidity and mortality [1].

Importantly, the biological and behavioural processes that shape ageing trajectories begin long before old age itself. Patterns of physical inactivity, suboptimal diet quality, and psychosocial stress tend to cluster early in adult life and may silently shape long-term metabolic, cardiovascular, and functional risk. Recent large-scale observational data in young adults compellingly illustrate how these lifestyles components coexist and reinforce one another, while also revealing that the majority of individuals are aware of the need for change but struggle to translate this awareness into sustained behavioural improvement (contribution 1). This life-course perspective provides a natural conceptual frame for the present Special Issue, which brings together contributions spanning molecular mechanisms, clinical studies, and population-level observations.

A recurring theme emerging from this Special Issue is that the relationship between nutrition, physical activity, and ageing is not merely additive, but fundamentally synergistic. Diet shapes metabolic and inflammatory tone, while movement modifies body composition, mitochondrial function, insulin sensitivity, and resilience to physiological stress [4]. Collectively, these processes determine vulnerability to frailty, sarcopenia, disability, and cardiometabolic disease [5]. Several contributions highlight, from different angles, that muscle health in ageing is not simply a matter of preserving mass, but of maintaining metabolic quality and functional efficiency. Interventions such as medium-chain triglyceride supplementation appear capable of shifting skeletal muscle substrate utilization and improving muscle quality without necessarily increasing muscle size, thereby offering a plausible mechanistic explanation for previously observed benefits in gait stability and balance in older adults (contribution 2). Simultaneously, observational data in older populations reinforce the central role of adequate protein intake in maintaining muscle mass, while also suggesting that the nutritional determinants of muscle health may be more complex and more strongly modulated by lifestyle factors in women than in men (contribution 3). Complementing this perspective, evidence synthesized on nitrate-rich beetroot supplementation suggests that nutritional modulation of vascular and muscular energetics can translate into meaningful improvements in physical performance, particularly in non-elite or ageing populations, even if cognitive benefits remain less consistent (contribution 4).

Beyond the musculoskeletal system, the contributions in this Issue also converge on the importance of low-grade inflammation, oxidative stress, and metabolic resilience as core features of biological ageing. Even in apparently healthy older adults, inflammatory and vascular risk markers are frequently elevated, reflecting the well-described phenomenon of “inflammaging” [6]. Notably, targeted, polyphenol-rich nutritional interventions appear capable of attenuating this low-grade inflammatory burden and improving vascular parameters over relatively short timeframes, supporting the concept that diet can modulate ageing-related pathophysiology even before disease becomes clinically apparent (contribution 5). At the other end of the age spectrum, data in physically active adolescents show that antioxidant status and redox balance are influenced more by habitual dietary patterns and nutrient timing than by single bouts of exercise. This illustrates the complexity and context-dependence of redox regulation and cautions against overly simplistic interpretations of antioxidant supplementation or acute exercise effects (contribution 6). By acting together, these findings reinforce the view that nutrition acts not merely as fuel, but as a regulator of inflammatory tone and stress adaptation capacity.

Several contributions also extend the discussion from functional outcomes to more fundamental mechanisms of ageing. Experimental evidence from animal models indicates that dietary interventions can influence genomic stability, DNA repair capacity, and mitochondrial function processes that occupy a central position in contemporary theories of ageing biology [7]. While caloric- and dietary restriction remain the most robust experimental strategies in this field, a broader spectrum of nutritional and metabolic interventions is now being explored, converging on the idea that metabolic–genomic crosstalk represents a critical interface between lifestyle and the biology of ageing (contribution 7) [8]. At the same time, the translational dimension of these insights should not be underestimated. The example of the Mediterranean diet in chronic inflammatory diseases shows that even the most biologically plausible and evidence-supported dietary patterns require careful adaptation to clinical context, patient preferences, and long-term feasibility in order to achieve durable benefits in real-world settings (contribution 8).

As a whole, the contributions assembled in this Special Issue offer a coherent and nuanced picture of how diet and physical activity shape ageing trajectories from early adulthood to old age and from molecular mechanisms to functional outcomes. A consistent message is that healthy longevity is not the product of any single intervention, nutrient, or training modality, but rather is the result of sustained, integrated lifestyle strategies that act on interconnected metabolic, inflammatory, vascular, and musculoskeletal pathways. Perhaps most importantly, these studies collectively remind us that the prevention of frailty, disability, and loss of independence cannot be postponed until old age itself, but must begin much earlier, through long-term investment in dietary quality and habitual physical activity [9]. Future research should therefore focus not only on refining mechanistic understanding, but also on developing scalable, pragmatic, and behaviourally informed approaches capable of delivering meaningful and lasting gains in healthspan at the population level.
